# The Functional Reorganization of Language Network Modules in Glioma Patients: New Insights From Resting State fMRI Study

**DOI:** 10.3389/fonc.2021.617179

**Published:** 2021-02-26

**Authors:** Lu Jin, Chuzhong Li, Yazhuo Zhang, Taoyang Yuan, Jianyou Ying, Zhentao Zuo, Songbai Gui

**Affiliations:** ^1^ Department of Neurosurgery, Beijing Tiantan Hospital, Capital Medical University, Beijing, China; ^2^ Beijing Neurosurgical Institute, Capital Medical University, Beijing, China; ^3^ State Key Laboratory of Brain and Cognitive Science, Institute of Biophysics, Chinese Academy of Sciences, Beijing, China

**Keywords:** language network, mean functional network connectivity, glioma, surgery, reorganization

## Abstract

**Background:**

Prior investigations of language functions have focused on the response profiles of particular brain regions. However, the specialized and static view of language processing does not explain numerous observations of functional recovery following brain surgery. To investigate the dynamic alterations of functional connectivity (FC) within language network (LN) in glioma patients, we explored a new flexible model based on the neuroscientific hypothesis of core-periphery organization in LN.

**Methods:**

Group-level LN mapping was determined from 109 glioma patients and forty-two healthy controls (HCs) using independent component analysis (ICA). FC and mean network connectivity (mNC: l/rFCw, FCb, and FCg) were compared between patients and HCs. Correlations between mNC and tumor volume (TV) were calculated.

**Results:**

We identified ten separate LN modules from ICA. Compared to HCs, glioma patients showed a significant reduction in language network functional connectivity (LNFC), with a distinct pattern modulated by tumor position. Left hemisphere gliomas had a broader impact on FC than right hemisphere gliomas, with more reduced edges away from tumor sites (*p*=0.011). mNC analysis revealed a significant reduction in all indicators of FC except for lFCw in right hemisphere gliomas. These alterations were associated with TV in a double correlative relationship depending on the tumor position across hemispheres.

**Conclusion:**

Our findings emphasize the importance of considering the modulatory effects of core-periphery mechanisms from a network perspective. Preoperative evaluation of changes in LN caused by gliomas could provide the surgeon a reference to optimize resection while maintaining functional balance.

## Introduction

Traditionally, the posterior part of the left superior temporal gyrus (Wernicke’s area) and the rostral part of the left inferior frontal cortices (Broca’s area) have been associated with language comprehension and production and are classically designated as “eloquent” areas (1, 2). However, recent studies have revealed that additionally bilateral temporal, parietal, prefrontal, and putamen regions (3) and even the cerebellum (4) are involved in language processing, reflecting a large-scale network engaging in language comprehension and production distributed at both the cortical and subcortical levels. Within this distributed language processing system, the left hemisphere frontotemporal subnetwork is widely assumed to underpin language comprehension in the key combinatorial language domains of grammatical computations and semantic operations (5), while the right hemisphere subnetwork contributes to linguistic working memory capacity (6, 7). Together, these findings indicate that the language system is best considered a core-periphery model regulated by a domain-general and domain-specific subnetwork (8), an approach that has been proven to be constructive in understanding the functional architecture of language (9).

As the language network strikes a balance between integration and segregation among functionally specialized brain regions, we hypothesized that distinct structural damage would lead to different maladaptation and reorganization of inter versus intra-hemisphere connectivity due to their distinct roles in the network. Compared with task-based fMRI, resting-state fMRI obviates task compliance from patients, allows the parallel assessment of functional networks. Abnormal communications within brain functional network has been reported in series of brain lesion studies using resting-state fMRI functional connectivity (10, 11), however, the degree to which lesion topography (sizes, locations) accounts for the variability of functional connectivity across different modules is mostly unknown.

Thus, we subdivided the language-related regions into four subsystems, the left frontal module, the left temporal module, and their contralateral homologues (12). Then, we performed ICA to identify LN maps at rest in the presence of structural brain damage caused by gliomas located in the four subsystems, and compared FC and mNC among LN modules between glioma patients and demographically matched HCs. Finally, the possible correlations between TV and mNC patterns in glioma patients were assessed.

## Materials and Methods

### Participants

112 glioma patients were recruited from our Department of Neurosurgery, and forty-two demographically matched HCs were included in this study. These patients were selected from a pool of database collected between December 2016 – November 2017. The patient selection flowchart can be seen in **Supplementary Figure S1**. All participants met the following criteria (1): Patients with a glioma affecting one of the language subsystem areas; (2) No symptoms of motor impairment; (3) Right hand according to the Edinburgh Inventory (13); (4) No history of brain surgery and psychiatric illness. Corresponding to the glioma locations in the four subsystems of language-related areas, we categorized the patients into four subgroups, the left frontal glioma subgroup (LFG), left temporal glioma subgroup (LTG), right frontal glioma subgroup (RFG), and right temporal glioma subgroup (RTG), to study the effect of lesion topography on language network functional connectivity (LNFC). This study was approved by the ethics committee of Beijing Tiantan Hospital, and written informed consent was obtained from all participants.

All participants were assessed by an experienced surgeon (J.Y.Y.) to identify the general psychological status on the day of MRI. Specifically, we assessed cognitive capability by the Mini Mental State Examination (MMSE), and motor and mental statuses were assessed with the Karnofsky Performance Scale (KPS) (14), Beck Depression Inventory (BDI) (15).

### Imaging Protocols

We acquired whole-brain fMRI data on a Siemens Prisma 3.0 Tesla scanner (Siemens Healthineers, Erlangen, Germany) with a 20-channel head coil. During the scanning, subjects were asked to relax and close their eyes. Three types of images were collected: (i) High-resolution 3D T1-weighted images; (ii) Resting-state fMR images; and (iii) T2-weighted images. The 3D T1-weighted sagittal images were acquired with a magnetism-prepared rapid acquired with gradient echo (MPRAGE) sequence: 192 slices, acquisition time = 7.4 min; acquisition matrix = 256 x 256, slice thickness/gap = 1/0 mm, TI/TR/TE = 900/2300/2.3 ms, flip angle = 8 deg, and field of view (FOV) = 256 x 256 mm^2^. The rs-fMRI data were acquired using an echo-planar image sequence: 30 axial slices, acquisition matrix = 64 x 64, slice thickness/gap = 5/0.5 mm, repetition time = 2,000 ms, echo time = 30 ms, and FOV = 192 x 192 mm^2^. The T2 images were acquired with a turbo-spin echo (TSE) sequence along the axial plane (33 axial slices; matrix size = 448 x 406, slice thickness/gap = 3/0.9 mm, repetition time = 5,000 ms, echo time = 105 ms, flip angle = 150 deg, and FOV = 220 x 199 mm^2^).

### Lesion Mapping

**Figure 1:**
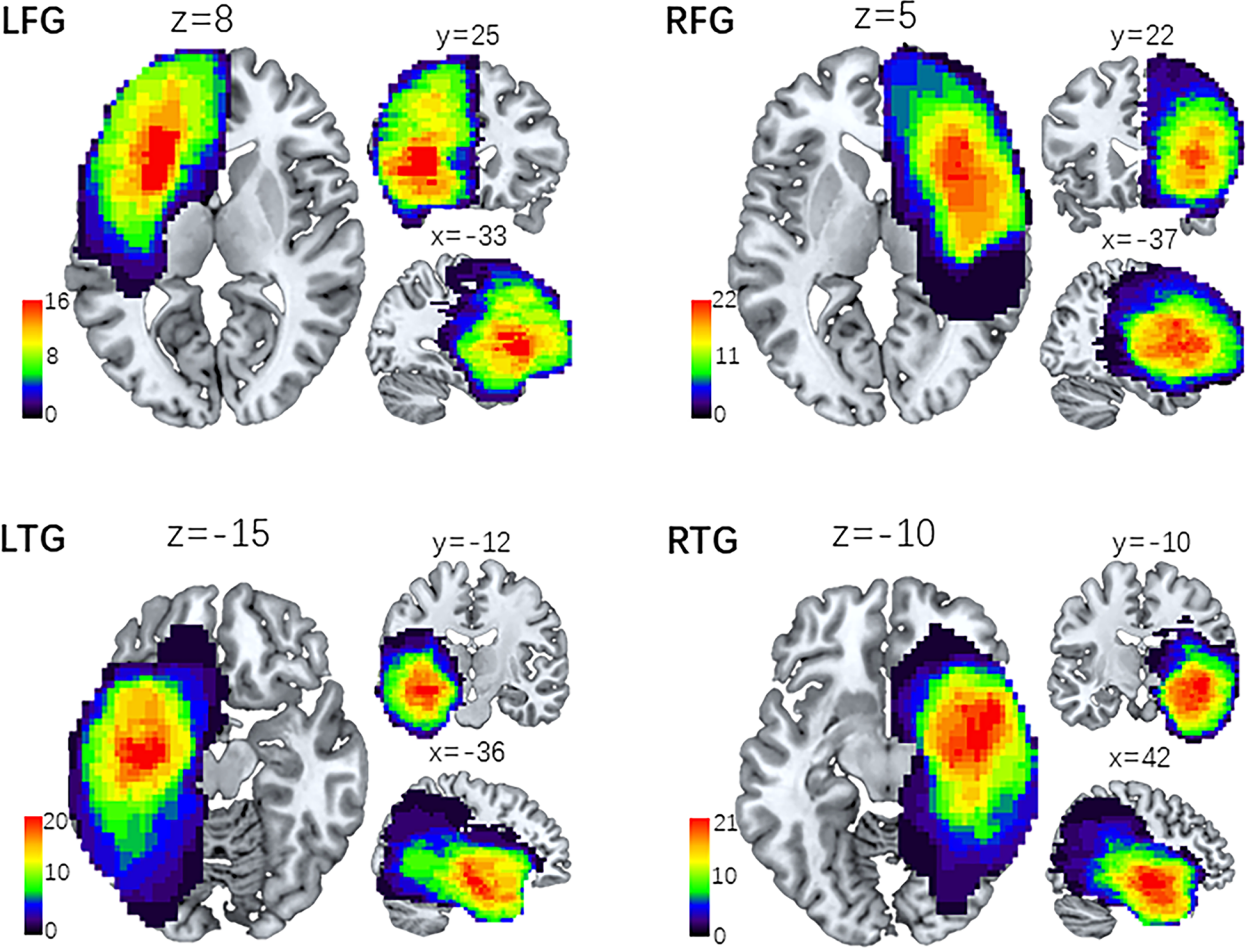
Lesion overlap maps of four subgroups of patients. Lesion distributive density maps are obtained by stacking together the manually traced tumor volume of interest (VOI). Then, the four groups of lesion maps are overlapped onto the MNI template separately. The coordinates (x, y, z) represent three dimensional positions on the standard template, and the color bar indicates the number of VOI in a given voxel. LFG, left frontal glioma; LTG, left temporal glioma; RFG, right frontal glioma; RTG, right temporal glioma.

To define the lesion feature of each patient anatomically, we first coregistered and resliced the T2 images to the native space of the averaged 3D images with the trilinear interpolation method in SPM12 (SPM12; Wellcome Department of Imaging Neuroscience, University College London, UK). Then, the images were normalized to the Montreal Neurological Institute (MNI) template with a spatial resolution of 1 × 1 × 1 mm. The resulting images were manually traced by a senior neurosurgeon (L.J.) with MRIcron software (http://people.cas.sc.edu/rorden/mricron.html). A volume of interest (VOI) was created for each patient. Delineation of the affected volume was based on the same criterion for all glioma patients: the entire hyperintense portion of the coregistered 3D T2-weighted images. A neurologist (T.Y.Y.) reviewed all segmentations a second time, paying special attention to the borders of the lesions and the degree of white matter disease. Then, we used the MATLAB script get_totals provided by Ridgway (www.cs.ucl.ac.uk/staff/g.ridgway/vbm/get_totals.m.) to obtain the tumor volume (TV) of every patient. Finally, patients’ tumor masks of the four subgroups were separately stacked together to construct overlapping tumor images, which are displayed in **Figure 1**.

### Image Preprocessing

Preprocessing was conducted using the DPABI toolbox (http://rfmri.org/dpabi). The first 10 volumes were discarded to allow for saturation effects and magnetization equilibrium. Then, the remaining images were slice-timing corrected, motion corrected, aligned with the anatomical scan, normalized to the MNI space, resampled to 3.0 mm^3^, and spatially smoothed with a 4-mm FWHM Gaussian kernel. The linear trend was removed for the time series of each voxel, and several nuisance signals, including Friston’s 24 head motion parameter, white matter and cerebrospinal fluid signals, were regressed out. Notably, we did not regress out the global signal because this procedure remains controversial. Finally, temporal bandpass filtering (0.01–0.08 Hz) was applied to the time courses. Of the 112 patients, three were excluded for excessive head motion (exclusion criteria: 3.0 mm and 3.0 degrees in maximum head motion). Finally, 109 glioma patients and 42 HCs were included, and the demographic data and clinical features of all participants are summarized in **Table 1**. Statistical analyses were conducted using SPSS (IBM, CA, USA), with the alpha level set at *p* < 0.05 for all tests.

**Table 1:** Sample demographic and clinical characteristics.

Variable	LFG(27)	LTG(26)	RFG(29)	RTG(27)	HC(42)	F/χ^2^	*P*
Age (mean ± SD, y)	45.04 ± 12.82	42.35 ± 11.53	40.76 ± 12.68	43.44 ± 13.93	39.55 ± 9.96	0.842	0.501^a^
Gender (M/F)	15/12	10/16	15/14	18/9	24/18	4.524	0.340^b^
Education (mean ± SD, y)	11.37 ± 3.58	13.12 ± 3.99	12.61 ± 3.78	11.93 ± 4.70	13.71 ± 3.79	1.776	0.137^a^
Tumor volume (mean ± SD, cm^3^)	106.6 ± 64.60	85.58 ± 44.69	110.7 ± 67.60	98.16 ± 66.77	–	0.866	0.461^a^
MMSE (mean ± SD)	28.11 ± 1.78	27.00 ± 3.62	27.41 ± 2.73	28.11 ± 2.61	29.48 ± 1.11	5.345	0.0005^a^
Language score	8.89 ± 0.32	8.35 ± 1.16	8.62 ± 0.62	8.56 ± 0.80	8.83 ± 0.49	2.75	0.03^a^
BDI (mean ± SD)	3.82 ± 4.23	2.77 ± 4.07	4.57 ± 4.93	4.11 ± 4.36	2.50 ± 3.60	1.452	0.220^a^
KPS (mean ± SD)	82.59 ± 11.30	85.77 ± 8.09	81.38 ± 11.25	81.84 ± 14.86	100.0 ± 0.00	23.98	<0.0001^a^
Pathological types							
Low-grade glioma	15	13	15	12	–		
Anaplastic astrocytoma/oligodendroglioma	7	8	5	4	–	5.66	0.462^b^
Glioblastoma	5	5	9	11	–		
Pathological grades							
Low-grade glioma(WHO I/II)	15	13	15	12	–	0.692	0.875^b^
High-grade glioma(WHO III/IV)	12	13	14	15	–		

BDI, Beck depression inventory; F, female; HC, healthy control; KPS, Karnofsky performance scale; LFG, left frontal glioma; LTG, left temporal glioma; M, male; MMSE, Mini mental state examination; NA, not applicable; RFG, right frontal glioma; RTG, right temporal glioma; SD =standard deviation; TV, tumor volume; ^a^p-value obtained using one-way ANOVA; ^b^p-value obtained using Pearson’s chi-square test.

### Group ICA

We performed ICA using GIFT software (http://icatb.sourceforge.net/, version 1.3b) for MATLAB on all subjects. Thirty independent components (ICs) were created as a group-wide spatial map of the resting-state networks.

### LN Analysis

**Figure 2:**
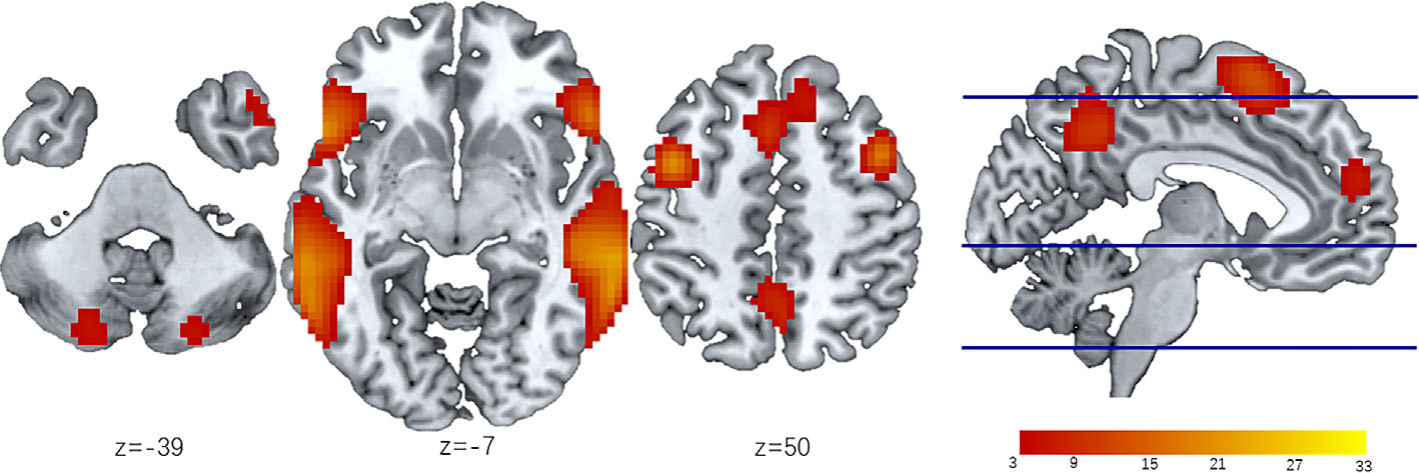
LN identified by ICA of all participants. The LN is extracted from 30 independent components obtained from Independent component analysis (ICA). Then the group level LN map is overlap onto the MNI template to create resting state spatial map of all participants (n=109). Eight separated cerebral clusters are obtained at a threshold of T > 5.0 and two cerebellar clusters are obtained at a threshold of T > 3.0. The z-coordinates represent axial positions on the standard space, and the color bar indicates the T value of LN modules. ICA, independent component analysis; LN, language network.

The IC showing the largest and most significant spatial correlation with the LN in the resting-state networks (RSNs) template (http://findlab.stanford.edu/functional_ROIs.htm) was selected by the software as the target network to study. Then, we obtained the mask of the LN consisting of eight separated cerebral clusters obtained at a threshold of T > 5.0 and two cerebellar clusters at a threshold of T > 3.0. This map was intended to identify the peak voxel coordinates of the LN for seed placement in the seed-based FC analysis. The LN maps identified by group-level ICA were shown in **Figure 2** and included the bilateral inferior frontal gyri (bi-IFG), bilateral middle frontal gyri (bi-MFG), bilateral superior temporal gyri (bi-STG), medial cingulate cortices (anterior, posterior), and bilateral cerebellar cortices.

### FC Analysis

A 6 mm spherical region of interest (ROI) was placed at the peak voxel of each LN cluster in the MNI template space. The LN ROIs and their MNI coordinates are delineated in **Table 2**. Mean signals extracted from the nodes were correlated with each other using Pearson’s correlation coefficient (r), resulting in a total of forty-five connectivity measurements (edges). These correlation coefficients were then converted to Z-scores using Fisher’s transformation. An independent two-sample t test was performed to compare all edge values in each patient subgroup with those in HCs. A threshold of one-tailed false discovery rate (FDR)-corrected *p* < 0.05 was applied to correct for multiple comparisons. These significantly reduced edges were classified as two different types: reduced edges connected with nodes within tumor located subsystems, which indicated connections directedly affected by tumor infiltration, and reduced edges connected between nodes in non-neoplastic modules, which suggested connections undirectedly disturbed by gliomas. Furthermore, the chi-square test was performed to analyze differences in the numbers of the two different edges between left and right hemisphere gliomas (LFG, LTG vs. RFG, RTG). *p* < 0.05 was considered statistically significant.

**Table 2:** List of the language network modules.

LN modules	Module size	Peak MNI Coordinates			T score
	(in voxels)	X	Y	Z	peak level
lIFG	218	-51	21	-9	10.93
lSTG	955	-57	-57	12	16.27
lMFG	136	-45	3	48	11.30
rIFG	127	54	24	-6	8.26
rSTG	1,149	60	-51	12	19.82
rMFG	104	45	9	45	9.86
l/rSFG	109	-3	12	57	7.85
l/rPC	133	0	-57	39	8.39
lCPL	92	-21	-78	-39	3.47
rCPL	43	21	-81	-42	3.17

The language network modules identified by ICA. The MNI coordinates of peak voxels are listed in the table. ICA, independent component analysis; LN, language network; lIFG, left inferior frontal gyrus; lSTG, left superior temporal gyrus; lMFG, left middle frontal gyrus; l/rSFG, left/right superior frontal gyrus; l/rPC, left/right precuneus; lCPL, left cerebellum posterior lobe; MNI, Montreal Neurological Institute. rIFG, right inferior frontal gyrus; rSTG, right superior temporal gyrus; rMFG, right middle frontal gyrus; rCPL, right cerebellum posterior lobe.

### NC Analysis

We selected homotopic ROIs (the same location on opposite hemispheres), including the bi-IFG, bi-MFG, and bi-STG, to balance the seed regions to obtain mNC representing ipsi/contralesional intrahemispheric and interhemispheric connectivity. The mNC values among the ROIs within the left and right hemispheres (intra/within-hemisphere, l/rFCw), among the ROIs between the left and right hemispheres (inter/between-hemisphere, FCb), and among all ROIs of the LN (global, FCg) were calculated by reducing each subject’s FC correlation matrix into a single variable, separately. NC analysis results were entered into one-way ANOVA. Finally, correlations between TV and mNC (l/rFCw, FCb, and FCg) were assessed by using Pearson coefficients.

### Data Availability

Anonymized data and data analysis pipeline will be shared by request from any qualified investigator.

## Results

### Demographic Data

109 glioma patients and forty-two demographically matched HCs were studied (**Table 1**): LFG (15 males/12 females, mean age 45.04 ± 12.82 years); LTG (10 males/16 females, mean age 42.35 ± 11.53 years); RFG (15 males/14 females, mean age 40.76 ± 12.68 years); RTG (18 males/9 females, mean age 43.44 ± 13.93 years); and HCs (24 males/18 females, mean age 39.55 ± 9.96 years). There were no significant differences among the five groups in age [F (4, 146) = 0.84; *p* =0.50], the distribution of sex (χ^2^ = 4.52; *p* =0.34), or level of education [F (4, 146) = 1.77; *p* = 0.14]. Although language scores derived from MMSE sections demonstrated a significant difference among patient groups and HCs [F (4, 146) = 2.75; *p* =0.03], no remarkable difference was found among the four patient groups [F (3, 105) = 1.77; *p* = 0.09]. And the pathological types and grades of glioma also showed no difference (χ^2^ = 5.66, *p* =0.462; χ^2^ = 0.692, *p* =0.875).

### Reduced FC Patterns in Glioma Patients

**Figure 3:**
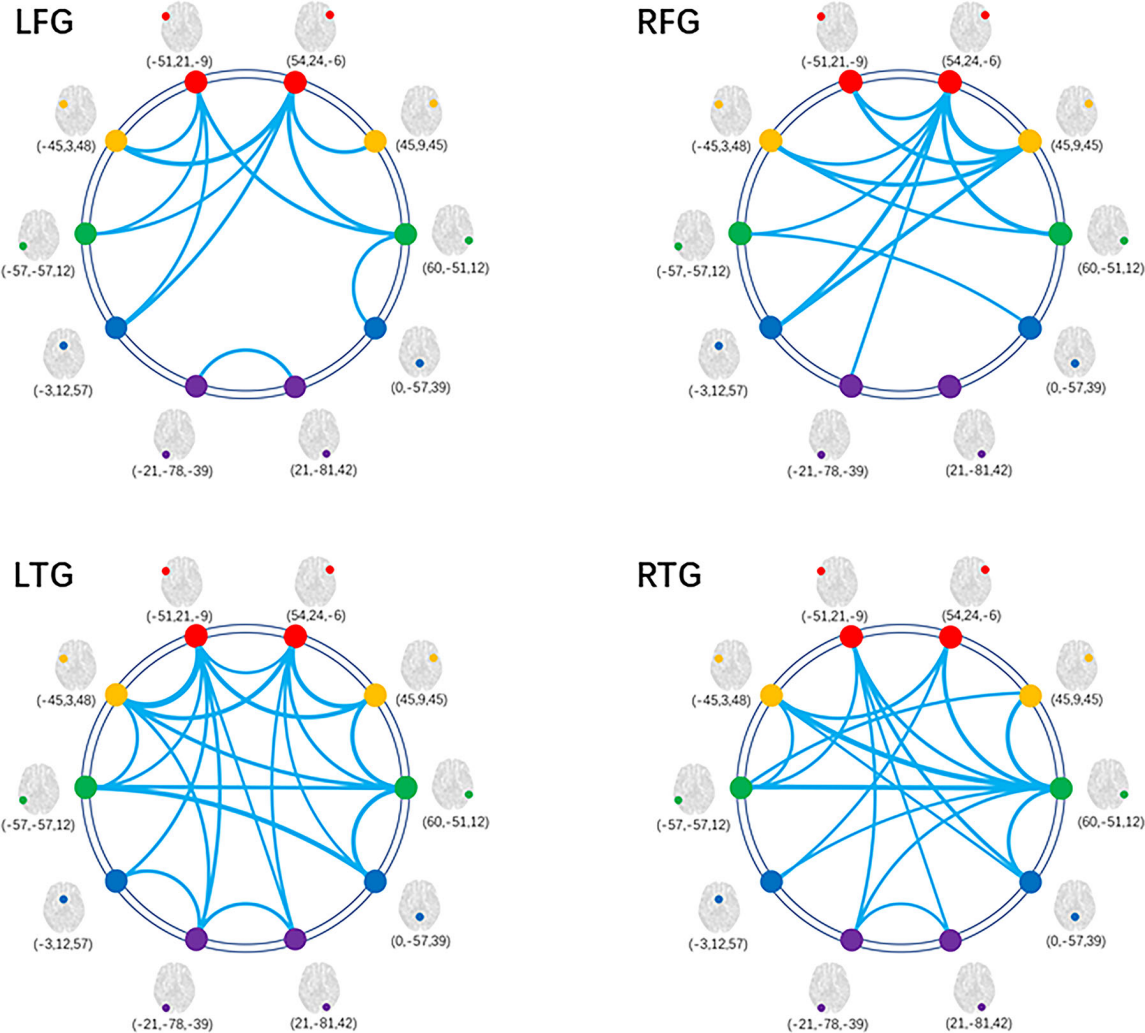
Decreased FC within LN for patient subgroups compared with HCs. Graphical presentation of the decreased FC within LN by comparing the four subgroups of patients with HCs separately. The thickness of lines is proportional to the absolute value of between-group T (one-tailed t test, FDR-corrected p < 0.05). FC, functional connectivity; HC, healthy control; LN, language network; LFG, left frontal glioma; LTG, left temporal glioma; RFG, right frontal glioma; RTG, right temporal glioma.

In glioma patients, we found significantly reduced FC of LN in comparison with HCs. **Figure 3** shows the distribution of FC edges with a significant reduction for the four subgroups (detailed results of the T values can be seen in the **Supplementary material, Table S1**). More importantly, we found that the left hemisphere lesions (LFG, LTG) caused much more severe disturbance to the whole-brain LN topology than the right hemisphere lesions (RFG, RTG) by comparing the numbers of reduced edges connected with nodes within tumor located subsystems (directly affected by gliomas) with edges connected between nodes in non-neoplastic modules (undirectedly disturbed by gliomas) (χ^2^ = 6.399, *p*=0.011, **Supplementary Results, Table S2**).

### Reduced NC at the Hemisphere Level

**Figure 4:**
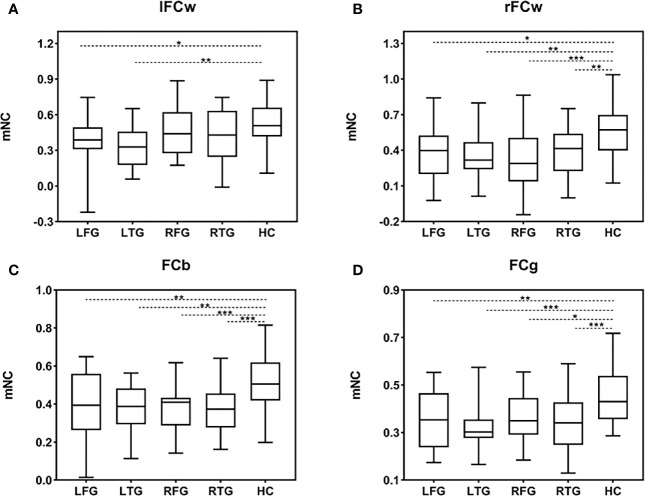
mNC differences among the four subgroups of patients and HCs. **(A–D)** Group differences in mNC (l/rFCw, FCb, and FCg) among the four subgroups of patients and HCs. Significant mNC differences are observed between the four subgroups of patients and HCs except for lFCw in the groups of RFG and RTG. Post hoc analysis shows that there is no difference in any aspect of mNC among the four subgroups of patients. Error bars represent one standard error of the mean (SEM). (*p < 0.05; **p < 0.01; ***p < 0.001). FCb, inter/between-hemisphere functional connectivity; FCg, global functional connectivity; HC, healthy control; LFG, left frontal glioma; LTG, left temporal glioma; lFCw, left intra/within-hemisphere functional connectivity; mNC, mean network connectivity; rFCw, right intra/within-hemisphere functional connectivity; RFG, right frontal glioma; RTG, right temporal glioma.

Having demonstrated the single-edge FC decrease in the LN in all subgroups, we further studied alterations in FC at the hemisphere level. One-way ANOVA was separately performed for mNC (l/rFCw, FCb, and FCg) among the four subgroups of patients and HCs (**Figure 4**). Specifically, in terms of FCb and FCg, Tukey’s test for multiple comparisons suggested significant differences between the HC and all four tumor subgroups (FCb: LFG *p*=0.003, LTG p=0.001, RFG *p*<0.001, RTG *p*<0.001, **Figure 4C**; FCg: LFG *p*=0.005, LTG p<0.001, RFG *p*=0.014, RTG *p*<0.001, **Figure 4D**). Interestingly, *post hoc* analysis confirmed that patients with left hemisphere lesions exhibited a simultaneous decrease in bilateral FCw (l/rFCw) compared with HCs (LFG: lFCw *p*=0.011, rFCw *p*=0.014; LTG: lFCw *p*=0.002, rFCw *p*=0.002, **Figures 4A, B**), while patients with right hemisphere lesions only exhibited a significant difference in ipsilesional FCw (rFCw) (RFG: lFCw *p*=0.760, rFCw *p*<0.001; RTG: lFCw *p*=0.200, rFCw *p*=0.007, **Figures 4A, B**). There was no difference in any aspect of mNC among the four subgroups of patients.

In addition, we did not find a remarkable difference between lFCw and rFCw in HCs using the paired t test [t(41)=0.893, *p*=0.377, **Supplementary Results, Figure S2**], indicating that the strength of LNFC between the left and right hemispheres is almost equivalent under normal circumstances. Based on this result, we then explored whether there is a difference in ipsilesional versus contralesional FC (ipsi-FCw, con-FCw) using repeated measures ANOVA with FCw as a within-subject factor and group as a between-subject factor. We found a main effect of FCw [F(1,109)=4.98, *p*=0.028], indicating that ipsilesional FC (mean=0.35) decreased, on average, much more significantly than contralesional FC (mean=0.41) for all glioma patients. Second, when considering the l/rFCw of each subgroup separately, there was a significant difference only in the RFG subgroup (paired t test: LFG *p*=0.564; LTG *p*=0.743; RFG *p*=0.018; RTG *p*=0.396; **Supplementary Results, Figure S3**)

### Association of LNFC With Glioma Topography

**Figure 5:**
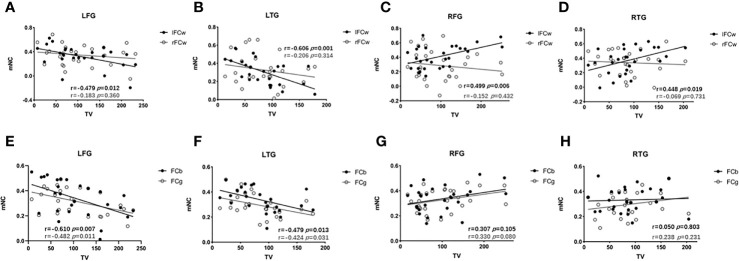
Associations between mNC and TV. Associations between mNC (l/rFCw, FCb, and FCg) and lesion volume. For LFG and LTG, TV is negatively correlated with ipsilesional (lFCw: LFG, R_27_=-0.479, *p*=0.012; LTG, R_26_=-0.606, *p*=0.001, **A, B**), interhemispheric (FCb: LFG, R_27_=-0.510, *p*=0.007; LTG, R_26_=-0.479, *p*=0.013, **E, F**), and global (FCg: LFG, R_27_=-0.482, *p*=0.011; LTG, R_26_=-0.424, *p*=0.031, **(E, F)**; while, for the RFG and RTG, positive correlations are found between lFCw and TV (lFCw: RFG, R_29_ = 0.499, *p*=0.006; RTG, R_27_ = 0.448, *p*=0.019; **(C, D)** and there are no remarkable negative correlations between TV and the other types of mNC (rFCw: RFG, R29 = -0.152, p=0.432, RTG, R27 = -0.069, p=0.731, C, D; FCb: RFG, R29 = 0.307, p=0.105, RTG, R27 = 0.050, p=0.803; FCg: RFG, R29 = 0.330, p=0.080, RTG, R27 = 0.238, p=0.231, **G, H**). FCb, inter/between-hemisphere functional connectivity; FCg, global functional connectivity; lFCw, left intra/within-hemisphere functional connectivity; LFG, left frontal glioma; LTG, left temporal glioma; mNC, mean network connectivity; rFCw, right intra/within-hemisphere functional connectivity; RFG, right frontal glioma; RTG, right temporal glioma; TV, tumor volume.

Next, we investigated the extent to which lesion topography explained the decrease in FC within LN in glioma patients. For the four subgroups of patients, we separately tested the relationship between brain lesions and mNC (l/rFCw, FCb, and FCg) using a univariate correlation with TV. For left hemisphere lesions (LFG, LTG), TV was negatively correlated with ipsilesional (lFCw: LFG, R_27_=-0.479, *p*=0.012; LTG, R_26_=-0.606, *p*=0.001, **Figures 5A, B**), interhemispheric (FCb: LFG, R_27_=-0.510, *p*=0.007; LTG, R_26_=-0.479, *p*=0.013, **Figures 5E, F**), and global (FCg: LFG, R_27_=-0.482, *p*=0.011; LTG, R_26_=-0.424, *p*=0.031, **Figures 5E, F**) FC but not with contralesional FC (rFCw: LFG, R_27_=-0.183, *p*=0.360; LTG, R_26_=-0.206, *p*=0.314, **Figures 5A, B**). However, in regard to the RFG and RTG subgroups, there were no remarkable negative correlations between TV and the four types of mNC. Interestingly, we found that TV was positively correlated with contralesional intrahemispheric FC (lFCw: RFG, R29 = 0.499, p=0.006; RTG, R27 = 0.448, p=0.019; **Figures 5C, D**). These contradictory results may suggest that tumors located in different language-related areas can reorganize the LN in different manners following the development of gliomas. And the strengths of reorganized mNC can be predicted by lesion volumes, but the prediction was not improved by adding information about lesion topography (pathology, grades). More details can be seen in the Supplementary material (**Supplementary Results, Table S3**).

## Discussion

In this study, we identified robust changes in LN synchrony (measured with rs-fMRI) in the setting of gliomas regardless of the specific functional-defined language areas that the tumor was involved with. However, the reduced pattern in LNFC following glioma development was modulated by tumor position. Gliomas in the left hemisphere had a broader impact on LN than gliomas in the right hemisphere, usually with weak connections between nodes located in non-neoplastic modules. Compared with HCs, mNC (l/rFCw, FCb, and FCg) showed a significant reduction in glioma patients (except for lFCw in the right hemisphere glioma subgroups [RFG, RTG]). In addition, mNC has a different correlative relationship with TV depending on the tumor position across hemispheres. These results agree with the proposal that language processing is the product of dynamic interactions among domain-specific and domain-general brain regions (16, 17); therefore, lesions in different eloquent areas have different impacts on the LN.

It is important to note that although the gliomas of the four subgroups led to a similarly widespread decrease in LNFC, their functional profiles varied considerably. In lesion-functional topography studies, one of the key methods often used to determine the importance of an area in network communication is to examine how much the network properties change if a given area is damaged (18). Core regions, also known as lesion hubs under disease status, have the greatest contribution to the functional network and are highly indispensable in the healthy network systems. Damage to these areas can have a fatally widespread effect on the transmission of information throughout the system (19, 20). Our study found that left frontotemporal subsystem gliomas strongly affected global LN communication, with the overwhelming majority of reduced edges away from the lesion sites, indicating that the transmission of information among remote modules in LN has been significantly disturbed. However, for right frontotemporal subsystem gliomas, most of the affected edges were connected with lesion sites, highlighting that disturbance to the right frontotemporal subsystems induced limited impacts on the whole network topology. With voxel-based lesion symptom mapping in mind, our study supports the hypothesis that both left frontal and temporal subsystems are the “core” regions of the LN; on the other hand, right frontal and temporal subsystems are the “periphery” regions (16, 17, 21).

From a core-periphery linguistic point of view, inter- and intrahemispheric FC was studied to identify specific alterations in mNC in the presence of gliomas. We found that the LFG and LTG subgroups demonstrated a consistent pattern in which mNC (l/rFCw, FCb, and FCg) decreased significantly in comparison with HCs. Moreover, decreased mNC between the two subgroups suggested no significant differences among the four types of mNC. By contrast, patients with right hemispheric gliomas (RFG, RTG) did not exhibit a remarkable decrease in mNC in the left frontotemporal subnetwork (lFCw) compared with HCs. Thus, our subsequent hemisphere level studies suggested that the LN was implemented in the model of left-dominant organization, damage to which has a very large disturbance on the LN, not only in communication among the left frontotemporal subnetwork but also in the structurally intact right frontotemporal subnetwork that participates in language processing (22). Furthermore, given the different alterations of left frontotemporal subnetwork, lFCw may be considered a sensitive indicator for distinguishing the extent of LNFC reorganization in the context of gliomas before surgery.

Most previous studies have revealed that the left frontotemporal network is crucial and specialized for language functions (23, 24). Comprehension and repetition deficits are associated with left frontotemporal brain damage, and lesion-deficit studies have revealed that lesion volume and the fractional anisotropy (FA) value of subcortical tissue are significantly correlated with the severity of impairment in language behavior (25, 26). Furthermore, the right hemisphere contributes to linguistic processing and working memory, which are needed for normal speech comprehension dynamically (27). Pathologies on the right temporal and frontal cortex tend to have a selective effect on language performance (e.g., cognitive control, working memory, executive function) (7, 28). However, our combined bilateral glioma study confirmed that right hemisphere structural damage only diminished subnetwork level connections among the periphery (rFCw) rather than core modules (lFCw). Thus, some aspects of key language functions may still be preserved in right hemisphere glioma patients; however, their language systems could be less synchronized, diminishing their ability to adapt dynamically to language processing demands (29).

The neurological mechanism underlying FC reduction between LN modules following glioma remains unclear. A possible explanation is the neurovascular uncoupling (NVU) –the abnormal tumor neo-vasculature may arouse disturbances over neuronal metabolism and neurotransmitter, which would affect the consistency and synchrony of neural functional activities at the whole brain level (30–32). In line with this notion, our findings additionally highlighted that the disturbances of connectivity with LN were associated with pathological topographies, especially lesion volumes and sites. For left hemisphere lesions (LFG, LTG), mNC, including ipsilesional intrahemispheric (lFCw), interhemispheric (FCb), and global (FCg) FC, was negatively correlated with TV. However, for right hemisphere lesions (RFG, RTG), there were no negative correlations between TV and the four types of mNC. Notably, although right hemispheric gliomas did not exhibit a significant decrease in contralesional intrahemispheric FC (lFCw) compared with HCs, a positive correlation was demonstrated between TV and lFCw. These interesting results suggest that disrupted communication among modules of LN is a central feature of left hemisphere lesions. Conversely, for right hemisphere lesions, it was likely to exist a dynamic functional compensation among LN core modules associated with tumor progression, which reflect the reconfiguration mechanism of core-periphery subsystems to regions most capable of meeting linguistic operations.

Our study found that the human language system retains functional organization under a hierarchical network framework, the processing of which depends on bilateral frontotemporal language areas. Language deficits can arise not only from a pure disruption in functionally specialized cores but also from a disturbance of communication among the functionally interconnected remote modules within LN (33). As demonstrated by numerous functional imaging studies, language disorders are well predicted both by lesion topography and FC: the former’s primary principle is that specific language functions dominate in specific regions of the brain, and the latter is based on the theory that high-level cognitive function is embedded with the connections among a large-scale functional network. Importantly, in addition to lesion-behavior and FC-behavior mapping, our study provides a more fundamental association between lesion topography and LNFC on the basis of cognitive functional studies. Given recent evidence for network data to provide prognostic markers (34–36), particularly in relation to postsurgical language outcomes, it is therefore anticipated that a combination of core-periphery connectome models with brain lesion topography will further our understanding of associated language dysfunctions and the likelihood of recovery following an operation—a crucial factor in facilitating surgical strategies and improving the care of glioma patients.

## Conclusions

Our findings emphasize the importance of considering the modulatory effects of core-periphery mechanisms when considering language reconfiguration for glioma patients from a network perspective. Lesion-behavior and FC-behavior models are important predictors for language dysfunction. The correlative relationship of these two factors suggests that a much broader approach is needed to evaluate language functional reorganization following glioma development. Recent evidence indicates that language recovery after surgery in glioma patients is linked to the organization patterns of the presurgical language system (37). Therefore, our findings of LN core-periphery organizational forms may provide the surgeon a reference to optimize resection of gliomas while maintaining a functional balance as well as a unique opportunity to further our understanding of the language system.

## Data Availability Statement

The raw data supporting the conclusions of this article will be made available by the authors, without undue reservation.

## Ethics Statement

The studies involving human participants were reviewed and approved by the Ethics Committee of Beijing Tiantan Hospital. The patients/participants provided their written informed consent to participate in this study.

## Author Contributions

LJ: design and conceptualized study, analyzed the data, and drafted the manuscript for intellectual content. CL: design and conceptualized study. YZ: design and conceptualized study. TY: major role in the acquisition of data. JY: major role in the acquisition of data. ZZ: interpreted the data and revised the manuscript for intellectual content. SG: interpreted the data and revised the manuscript for intellectual content. All authors contributed to the article and approved the submitted version.

## Funding

Our study is funded by Beijing Municipal Science & Technology Commission (Grant No. Z171100000117002), Beijing Municipal Science & Technology Commission (Grant No. Z191100006619087), and National Natural Science Foundation of China (Nos. 61872020).

## Conflict of Interest

The authors declare that the research was conducted in the absence of any commercial or financial relationships that could be construed as a potential conflict of interest.
