# Supplementary material for: The Functional Reorganization of Language Network Modules in Glioma Patients: New Insights From Resting State fMRI Study
Source: Front Oncol. 2021 Feb 26;11:617179. doi: 10.3389/fonc.2021.617179 (PMC7953055; doi:10.3389/fonc.2021.617179)
Supplement: Supplementary file 1 [file DataSheet_1.docx]

**Supplementary Figure S1.** A presentation of patient selection flowchart

**
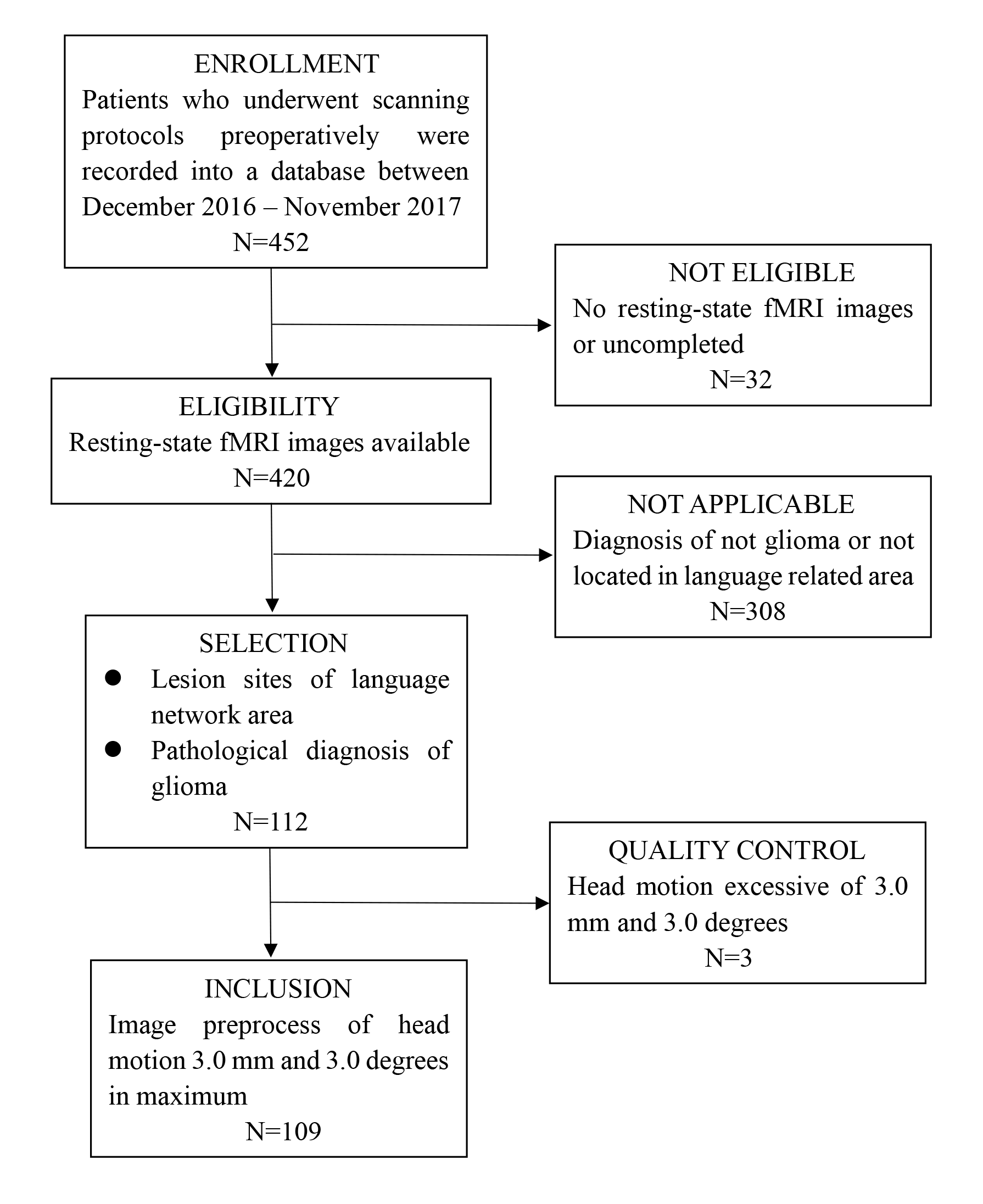
**

**Supplementary Figure S2.** Paired T test performed between lFCw and rFCw in HC


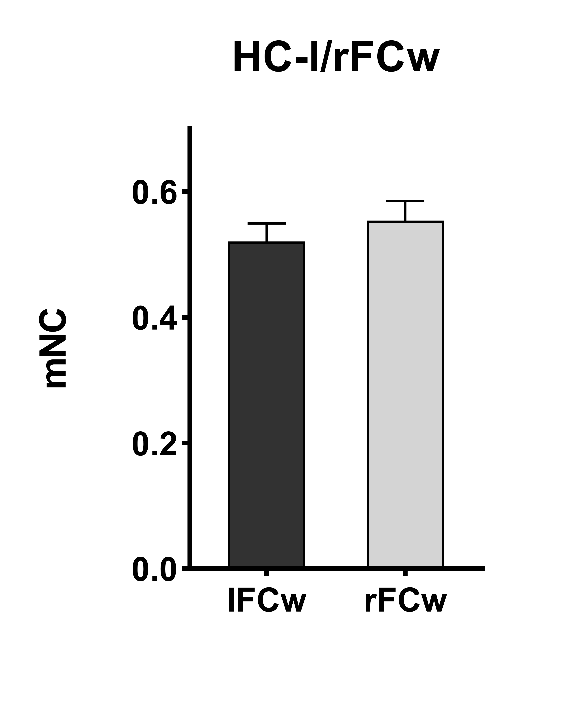


There was no remarkable difference between lFCw and rFCw in HC [t (41) = 0.893, *p* = 0.377]. mNC = mean network connectivity; lFCw = left intra/within-hemisphere functional connectivity; rFCw = right intra/within-hemisphere functional connectivity; HC = healthy control

**Supplementary Figure S3.** Comparison of mNC (l/rFCw) in each subgroup


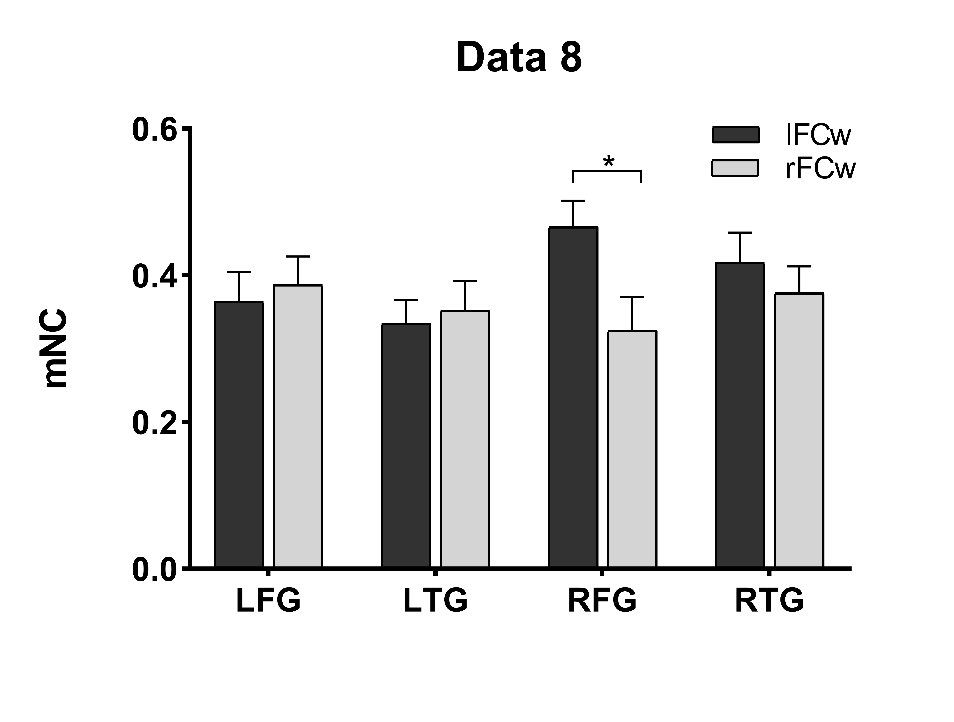


There was only a significant difference in the subgroup of RFG (paired t test: LFG *p* = 0.564; LTG *p* = 0.743; RFG *p* = 0.018; RTG *p* = 0.396). mNC = mean network connectivity; lFCw = left intra/within-hemisphere functional connectivity; rFCw = right intra/within-hemisphere functional connectivity; LFG = left frontal glioma; LTG = left temporal glioma; RFG = right frontal glioma; RTG = right temporal glioma

**Supplementary Table S1.** ROI pairs within LN that are significantly decreased in FC between subgroups of patients and HC

| **Node 1 (MNI coordinates)** | **Node 2 (MNI coordinates)** | **T** | ***p* value** |
| --- | --- | --- | --- |
| **LFG vs. HC** |  |  |  |
| rSTG (60, -51, 12) | rIFG (54, 24, -6) | -3.01 | 0.002 |
| rSTG (60, -51, 12) | lIFG (-51, 21, -9) | -2.95 | 0.002 |
| rSTG (60, -51, 12) | l/rPC (0, -57, 39) | -2.74 | 0.004 |
| lSTG (-57, -57,12) | rIFG (54, 24, -6) | -2.43 | 0.009 |
| lSTG (-57, -57,12) | lIFG (-51, 21, -9) | -2.52 | 0.007 |
| rIFG (54, 24, -6) | lMFG (-45, 3, 48) | -3.44 | 0.0005 |
| rIFG (54, 24, -6) | rMFG (45, 9, 45) | -2.93 | 0.002 |
| rIFG (54, 24, -6) | l/rSFG (-3, 12, 57) | -2.68 | 0.005 |
| lIFG (-51, 21, -9) | lMFG (-45, 3, 48) | -2.74 | 0.004 |
| lIFG (-51, 21, -9) | l/rSFG (-3, 12, 57) | -2.50 | 0.008 |
| lCPL (-21, -78, -39) | rCPL (21, -81, -42) | -2.82 | 0.003 |
| **LTG vs. HC** |  |  |  |
| rSTG (60, -51, 12) | lSTG (-57, -57,12) | -2.86 | 0.003 |
| rSTG (60, -51, 12) | rIFG (54, 24, -6) | -2.59 | 0.006 |
| rSTG (60, -51, 12) | l/rPC (0, -57, 39) | -3.42 | 0.0005 |
| rSTG (60, -51, 12) | lMFG (-45, 3, 48) | -2.86 | 0.003 |
| rSTG (60, -51, 12) | rMFG (45, 9, 45) | -3.22 | 0.001 |
| lIFG (-51, 21, -9) | lIFG (-51, 21, -9) | -2.46 | 0.008 |
| lIFG (-51, 21, -9) | l/rPC (0, -57, 39) | -3.68 | 0.0002 |
| lIFG (-51, 21, -9) | lMFG (-45, 3, 48) | -2.61 | 0.006 |
| rIFG (54, 24, -6) | lIFG (-51, 21, -9) | -2.27 | 0.013 |
| rIFG (54, 24, -6) | l/rPC (0, -57, 39) | -2.19 | 0.016 |
| rIFG (54, 24, -6) | lMFG (-45, 3, 48) | -3.26 | 0.0008 |
| rIFG (54, 24, -6) | rMFG (45, 9, 45) | -3.36 | 0.0006 |
| rIFG (54, 24, -6) | rSTG (60, -51, 12) | -2.32 | 0.012 |
| lIFG (-51, 21, -9) | lMFG (-45, 3, 48) | -4.46 | 0.0002 |
| lIFG (-51, 21, -9) | rMFG (45, 9, 45) | -3.31 | 0.0008 |
| lIFG (-51, 21, -9) | l/rSFG (-3, 12, 57) | -2.55 | 0.007 |
| lIFG (-51, 21, -9) | lSTG (-57, -57,12) | -3.16 | 0.001 |
| lIFG (-51, 21, -9) | rSTG (60, -51, 12) | -2.20 | 0.016 |
| lMFG (-45, 3, 48) | lSTG (-57, -57,12) | -2.50 | 0.008 |
| l/rSFG (-3, 12, 57) | lSTG (-57, -57,12) | -2.72 | 0.004 |
| lSTG (-57, -57,12) | rSTG (60, -51, 12) | -2.68 | 0.005 |
| **RFG vs. HC** |  |  |  |
| rSTG (60, -51, 12) | rIFG (54, 24, -6) | -3.32 | 0.0007 |
| rSTG (60, -51, 12) | lMFG (-45, 3, 48) | -2.36 | 0.011 |
| lSTG (-57, -57,12) | l/rPC (0, -57, 39) | -2.52 | 0.007 |
| rIFG (54, 24, -6) | lIFG (-51, 21, -9) | -2.82 | 0.003 |
| rIFG (54, 24, -6) | lMFG (-45, 3, 48) | -2.49 | 0.008 |
| rIFG (54, 24, -6) | rMFG (45, 9, 45) | -4.14 | 0.0005 |
| rIFG (54, 24, -6) | l/rSFG (-3, 12, 57) | -3.18 | 0.001 |
| rIFG (54, 24, -6) | lSTG (-57, -57,12) | -2.27 | 0.013 |
| lIFG (-51, 21, -9) | rMFG (45, 9, 45) | -3.19 | 0.001 |
| lMFG (-45, 3, 48) | rMFG (45, 9, 45) | -3.23 | 0.001 |
| rMFG (45, 9, 45) | l/rSFG (-3, 12, 57) | -2.99 | 0.002 |
| **RTG vs. HC** |  |  |  |
| rSTG (60, -51, 12) | lSTG (-57, -57,12) | -3.61 | 0.0003 |
| rSTG (60, -51, 12) | rIFG (54, 24, -6) | -3.25 | 0.0009 |
| rSTG (60, -51, 12) | lIFG (-51, 21, -9) | -2.66 | 0.005 |
| rSTG (60, -51, 12) | l/rPC (0, -57, 39) | -2.77 | 0.004 |
| rSTG (60, -51, 12) | lMFG (-45, 3, 48) | -3.86 | 0.0001 |
| rSTG (60, -51, 12) | rMFG (45, 9, 45) | -3.31 | 0.0007 |
| rSTG (60, -51, 12) | l/rSFG (-3, 12, 57) | -2.17 | 0.017 |
| rSTG (60, -51, 12) | lSTG (-57, -57,12) | -2.47 | 0.008 |
| lSTG (-57, -57,12) | lIFG (-51, 21, -9) | -2.28 | 0.013 |
| lSTG (-57, -57,12) | lMFG (-45, 3, 48) | -2.42 | 0.009 |
| lSTG (-57, -57,12) | rMFG (45, 9, 45) | -2.39 | 0.010 |
| rIFG (54, 24, -6) | lMFG (-45, 3, 48) | -2.67 | 0.005 |
| rIFG (54, 24, -6) | l/rSFG (-3, 12, 57) | -2.31 | 0.012 |
| lIFG (-51, 21, -9) | l/rPC (0, -57, 39) | -2.85 | 0.003 |
| lIFG (-51, 21, -9) | lSTG (-57, -57,12) | -2.37 | 0.010 |
| lIFG (-51, 21, -9) | rSTG (60, -51, 12) | -2.21 | 0.015 |
| l/rPC (0, -57, 39) | lMFG (-45, 3, 48) | -2.11 | 0.019 |
| lSTG (-57, -57,12) | rSTG (60, -51, 12) | -2.33 | 0.012 |

Both T values and corresponding *p* values are listed (FDR-corrected *p* < 0.05). LFG = left frontal glioma; LTG = left temporal glioma; RFG = right frontal glioma; RTG = right temporal glioma; HC = healthy control; lIFG = left inferior frontal gyrus; lSTG = left superior temporal gyrus; lMFG = left middle frontal gyrus; rIFG = right inferior frontal gyrus; rSTG = right superior temporal gyrus; rMFG = right middle frontal gyrus; l/rSFG = left/right superior frontal gyrus; l/rPC = left/right precuneus; lCPL = left cerebellum posterior lobe; rCPL = right cerebellum posterior lobe; MNI = Montreal Neurological Institute

| Connections (with tumor site) | | Connected | Unconnected | Total |
| --- | --- | --- | --- | --- |
| Hemisphere | Left | 9 | 23 | 32 |
|  | Right | 18 | 12 | 30 |
| Total | | 27 | 35 | 62 |

**Supplementary Table S2.** Numbers of significant reduced connections connected and unconnected with tumor sites in the left and right hemisphere glioma patients (χ2 = 6.399, *p* = 0.011)

**Supplementary Table S3.** Partial correlation analysis of mNC (l/rFCw, FCb, and FCg) with TV in combination with regression of other lesion topographies (pathology, grades)

| Subgroups |  | lFCw | rFCw | FCb | FCg |
| --- | --- | --- | --- | --- | --- |
| LFG | Correlation | -0.375 | -0.319 | -0.376 | -0.422 |
|  | Significance | 0.065 | 0.120 | 0.064 | 0.036 |
| LTG | Correlation | -0.545 | -0.379 | -0.422 | -0.453 |
|  | Significance | 0.006 | 0.068 | 0.040 | 0.026 |
| RFG | Correlation | 0.478 | -0.118 | 0.341 | 0.274 |
|  | Significance | 0.012 | 0.559 | 0.082 | 0.166 |
| RTG | Correlation | 0.621 | 0.046 | 0.178 | 0.298 |
|  | Significance | 0.001 | 0.828 | 0.393 | 0.147 |

Abbreviations: mNC = mean network connectivity; lFCw = left intra/within-hemisphere functional connectivity; rFCw = right intra/within-hemisphere functional connectivity; FCb = inter/between-hemisphere functional connectivity; FCg = global functional connectivity; TV = tumor volume; LFG = left frontal glioma; LTG = left temporal glioma; RFG = right frontal glioma; RTG = right temporal glioma
